# Rhenium Nanostructures Loaded into Amino-Functionalized Resin as a Nanocomposite Catalyst for Hydrogenation of 4-Nitrophenol and 4-Nitroaniline

**DOI:** 10.3390/polym13213796

**Published:** 2021-11-02

**Authors:** Piotr Cyganowski, Anna Dzimitrowicz, Piotr Jamroz, Dorota Jermakowicz-Bartkowiak, Pawel Pohl

**Affiliations:** 1 Department of Process Engineering and Technology of Polymer and Carbonaceous Materials, Faculty of Chemistry, Wroclaw University of Science and Technology, 50-370 Wroclaw, Poland; dorota.jermakowicz-bartkowiak@pwr.edu.pl; 2 Department of Analytical Chemistry and Chemical Metallurgy, Faculty of Chemistry, Wroclaw University of Science and Technology, 50-370 Wroclaw, Poland; anna.dzimitrowicz@pwr.edu.pl (A.D.); piotr.jamroz@pwr.edu.pl (P.J.); pawel.pohl@pwr.edu.pl (P.P.)

**Keywords:** polymeric nanocomposites, anion exchange resins, suspension copolymers, amines, 4-nitrophenol, 4-nitroaniline

## Abstract

The present work presents a new nanocomposite catalyst with rhenium nanostructures (ReNSs) for the catalytic hydrogenation of 4-nitrophenol and 4-nitroaniline. The catalyst, based on an anion exchange resin with functionality derived from 1,1′-carboimidazole, was obtained in the process involving anion exchange of ReO_4_^–^ ions followed by their reduction with NaBH_4_. The amino functionality present in the resin played a primary role in the stabilization of the resultant ReNSs, consisting of ≈1% (*w/w*) Re in the polymer mass. The synthesized and capped ReNSs were amorphous and had the average size of 3.45 ± 1.85 nm. Then, the obtained catalyst was used in a catalytic reduction of 4-nitrophenol (4-NP) and 4-nitroaniline (4-NA). Following the pseudo-first-order kinetics, 5 mg of the catalyst led to a 90% conversion of 4-NP with the mass-normalized rate constant (k_m1_) of 6.94 × 10^−3^ min^−1^ mg^−1^, while the corresponding value acquired for 4-NA was 7.2 × 10^−3^ min^−1^ mg^−1^, despite the trace amount of Re in the heterogenous catalyst. The obtained material was also conveniently reused.

## 1. Introduction

Nitroaromatic compounds (NARs) have been identified as one of the major chemicals revealing toxic, mutagenic, and carcinogenic character [1,2]. As a result of the above-mentioned health risk imposed by NARs, a strict control of environmental and health hazards arising from their occurrence is of a great concern all over the world.

One of the currently preferred approaches to the neutralization of NARs is their direct reduction to aromatic amines (AAMs) [3,4,5,6]. An industrial practice is to stoichiometrically reduce NARs with reducing agents, such as Na_2_S_2_O_4_, Fe, Sn, or Zn in NH_4_OH [3,4]. However, this process is recognized to be environmentally unsustainable. In turn, an alternative approach is used, with H_2_ and a metal oxide catalyst, but it leads to the formation of multiple by-products, whose further conversion to amines is yet another challenge [7]. Unfortunately, more selective catalysts, such as Co-Ru sulfides, yield poor reduction efficiencies and cause some additional contamination of the resultant AAMs [4]. As such, more attention has been paid to noble metals-based catalysts such as Au, Pt, and Pd. They have been turned out to be very effective and resulted in obtaining high yields of NARs conversion [3,5,6]. Unfortunately, these catalysts lack selectivity, leading to undesired interactions with reducible groups other than –NO_2_, i.e., carbonyls and others containing unsaturated bonds [3,5,6]. As a result of this, the scientific literature has been focused on applying metallic nanostructures as nanocatalysts (NCats) for the reduction of NARs [8]. Such NPs, derived from both precious as well as non-precious metals [3,8], are selective and preferably reduce –NO_2_ groups over other reducible moieties. Moreover, they offer a high stability and are useful for NARs reduction under mild conditions [8,9,10]. However, a practical application of metallic NPs is limited, as they concurrently reveal a tendency to aggregate and sediment [11]. A solution to this problem seems to be the synthesis of polymeric nanocomposites (pNCs) that contain metallic NPs. Such pNCs do not only limit the catalytic activity of metallic NPs but even enhance it, ensuring their stability and safety [12]. As such, more attention is put in the literature regarding the synthesis of NPs supported by an organic matter containing reductive moieties. This includes, e.g., the metal vapors synthesis (MVS) method using a metal scavenger [12,13], the hydrothermal method [14], as well as the reduction-coupled adsorption method occurring at a solid–liquid interface [15,16].

Based on the author’s own works [15,16], pNCs loaded with AuNPs facilitate the catalytic activity of these nanostructures in the catalytic reduction of 4-nitrophenol (4-NP). Moreover, the morphology of the polymeric matrix (suspension copolymers) enables an efficient re-use of the so-prepared catalysts, retaining the catalytic activity of AuNPs for over 11 cycles [15]. Hence, it is hypothesized that, similarly as in the case of AuNPs, a barely known nanomaterial, i.e., rhenium nanostructures (ReNSs) as active sites of pNCs-based NCats, would significantly boost the catalytic activity of such NCats toward NARs reduction. However, due to a rare occurrence and a high price, the literature lacks the information on ReNSs. There are only a few reports on ReNSs synthesis that include advanced physical processes [17,18,19,20,21]. It is also confirmed that ReNPs reveal an extraordinary catalytic activity outperforming this for AuNPs and NPs of platinum group metals (PGMs) in organic syntheses (e.g., oxidation of olefins) [18,22,23,24]. Although two examples are reported so far in which ReNSs-based NCats outperform other PGMs catalysts in the decomposition of NARs [25,26], none of these examples provides a solution to problems of heterogenous catalysis related to the stability of the NCat and a difficulty with its operation.

As a result of this, the premise of the present work was to synthesize a new pNC that would be based on ReNSs loaded directly within the structure of a suspension copolymer (in situ). It is hypothesized that such an approach would result in the synthesis of a heterogenous NCat, revealing an increased catalytic activity owed to the stable ReNSs presence. The procedure involved an anion exchange reaction between ReO_4_^–^ oxoanions and amino functionalities derived from 1,1′-carboimidazole present in the polymeric matrix. Then, Re (VII) ions were reduced, and the resultant ReNSs were precipitated within the polymer, forming a new pNC with ReNSs. Then, the so-obtained heterogenous NCat was employed in the catalytic process involving model reactions of 4-NP and 4-NA hydrogenation. It was expected that due to a unique catalytic activity of ReNSs, followed by their direct precipitation (in situ) within a polymeric matrix, the so-prepared NCats would outperform other heterogenous catalysts with metallic NPs, simultaneously ensuring the same or even better catalytic activity.

## 2. Experimental

### 2.1. Materials, Methods of Analyses, and Instrumentation

The reagents for synthesis of the polymeric matrix (anion exchange resin), i.e., vinylbenzyl chloride (VBC, 99%, mixture of 3- and 4-isomers), divinylbenzene (DVB, 80%), and 1,1′-carbonyldimidazole (99%, CIM), were acquired from MERCK (branch Poland). The monomers (VBC, DVB) were purified before their use by vacuum distillation. Ammonium perrhenate (VII) (NH_4_ReO_4_, >99%) and NaBH_4_ (>99%), used for loading the polymer with ReNSs, as well as reagents for the catalytic activity test, i.e., 4-NP (>99%) and 4-NA (>99%), were purchased in MERCK (branch Poland) and used as received. All other reagents and materials mentioned in this manuscript were acquired from Avantor Performance Materials Ltd. (Gliwice, Poland) and used without any pre-treatment. All experiments were carried out using RO water (Merck, Milipore, branch Poland).

Changes in the structure of pNC’s functionalities were verified by attenuated total reflectance Fourier transformation infrared spectroscopy (ATR-FTIR) using a JASCO FT-IR 4700 instrument (Santa Clara, CA, USA). The spectra were recorded using a diamond ATR attachment with resolution of 4 cm^−1^ and taking 64 scans for each sample. The concentration of ReNSs in the resultant pNC was determined by wet digesting its appropriate samples (in *aqua regia*, at 130 °C for 2.5 h) and analyzing the so-prepared sample solutions by inductively coupled plasma optical emission spectrometry (ICP-OES) (Agilent, Santa Clara, CA, USA); an Agilent instrument, model 5110, was used in this case. The morphology of the obtained pNC was assessed using scanning electron microscopy (SEM) (FEI Company, Hillsboro, OR, USA) with the aid of a FEI Helios NanoLab 450 HF instrument. The morphology of ReNSs loaded into the polymer was verified using transmission electron microscopy (TEM) (FEI Company, Hillsboro, OR, USA). All samples were prepared in the form of ultra-thin slices, which were placed on a Cu grid. Then, an FEI Tecnai G^2^X-TWIN instrument equipped with an energy-dispersive X-ray analyzer (FEI Company, Hillsboro, OR, USA) as well as a selected area energy diffraction (SAED) diffractometer was applied. UV-Vis spectrophotometry was used to monitor the catalytic hydrogenation of 4-NP and 4-NA; it was done by recording the corresponding spectra in the range of 200–600 nm using a Jasco V-530 (Easton, MD, USA) spectrophotometer. All measurements were independently repeated 3 times to get average values along with standard deviations (SDs).

### 2.2. Synthesis of Heterogenous NCat

**Figure 1:**
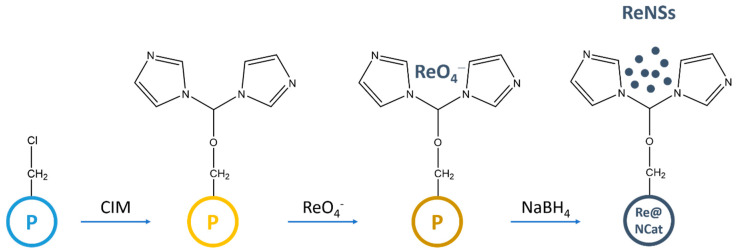
A simplified scheme of Re@NCat synthesis. The structure of the CIM functionality is proposed, based on the FT-IR analysis.

The polymeric base was obtained via the suspension copolymerization of VBC and DVB in the presence of toluene, poly(vinyl alcohol) as a suspension stabilizer and benzoyl peroxide as a free radical initiator, according to the procedure previously described in [27]. The resultant VBC-co-DVB copolymer was extracted into toluene and then dried. Afterwards, the obtained copolymer was introduced into dimethylformamide for 24 h (DMF, 10 mL per 1 g of the copolymer) to swell it before its modification. Further steps of the procedure are displayed in Figure 1. To carry out amino modification, CIM was added to the so-prepared mixture (5:1 ratio in respect to the Cl concentration in the VBC-co-DVB copolymer). The mixture of the polymeric matrix, solvent, and amine was agitated at ambient temperature using an orbital shaker for 14 days to enable a nucleophilic attack of CIM on –CH_2_Cl groups derived from VBC. After this, the resultant anion exchange resin was filtered, excessively washed with water, and subsequently conditioned using 1 mol L^−1^ HCl and NaOH solutions. Finally, the polymer was washed with 0.001 mol L^−1^ HCl. Then, 0.1 g of the so-prepared resin was contacted with 20 mL of a NH_4_ReO_4_ solution (2000 mg L^−1^ of Re in 0.1 mol L^−1^ HCl) for 24 h. After that, the Re (VII)-saturated resin was separated by filtration, washed with water, and put into 20 mL of water purged with N_2_. Next, 5 mL of a 0.1 mol L^−1^ NaBH_4_ solution was added to the so-prepared mixture. Immediately after this, the present polymeric beads changed their color from yellow to black, indicating a reduction of ReO_4_^−^ oxyanions. Although the process was rapid, the reaction mixture was agitated for 24 h with the aid of an orbital shaker to finalize the reduction process and obtain the Re@NCat material.

### 2.3. Catalytic Activity

To evaluate the Re@NCat catalytic activity towards the hydrogenation of NARs, 4-NP and 4-NA reductions were carried out as model reactions. The process was traced using UV/Vis absorption spectrophotometry. Accordingly, a 3 mL portion of a 0.1 mmol L^−1^ 4-NP (or 4-NA) solution was introduced into a quartz cuvette, and the maximum absorbance (A) at a specified wavelength (λ_max_, 318 nm for 4-NP and 380 nm for 4-NA) was read out. Next, 0.1 mL of a 0.1 mol L^−1^ NaBH_4_ solution was added to the cuvette with the 4-NP solution. After this, the absorbance at λ_max_ of red-shifted 4-NP was read at 400 nm, and the absorbance at λ_max_ of unaltered 4-NA was read at 380 nm. Afterwards, 5 or 50 mg of the Re@NCat was added to the mixture, and the changes of the absorbance at both specified λ_max_ were measured. Then, the acquired data on the initial absorbance (*A*_0_) and the absorbance at a time *t* (*A_t_*) were re-calculated using the pseudo-first-order kinetics, and the *lnA_t_/A*_0_ vs. *t* plot was drawn. The value of the slope of this plot was used to get the pseudo-first-order rate constant (*k*_1_, min^−1^). Then, the *k*_1_ value was re-calculated to the mass-normalized rate constant (*k_m_*_1_, min^−1^ mg^−1^), and this enabled comparing the catalytic activity of the synthesized catalyst with that found in the literature for other catalysts.

## 3. Results and Discussion

### 3.1. Synthesis of the Nanocomposite with ReNSs

**Figure 2:**
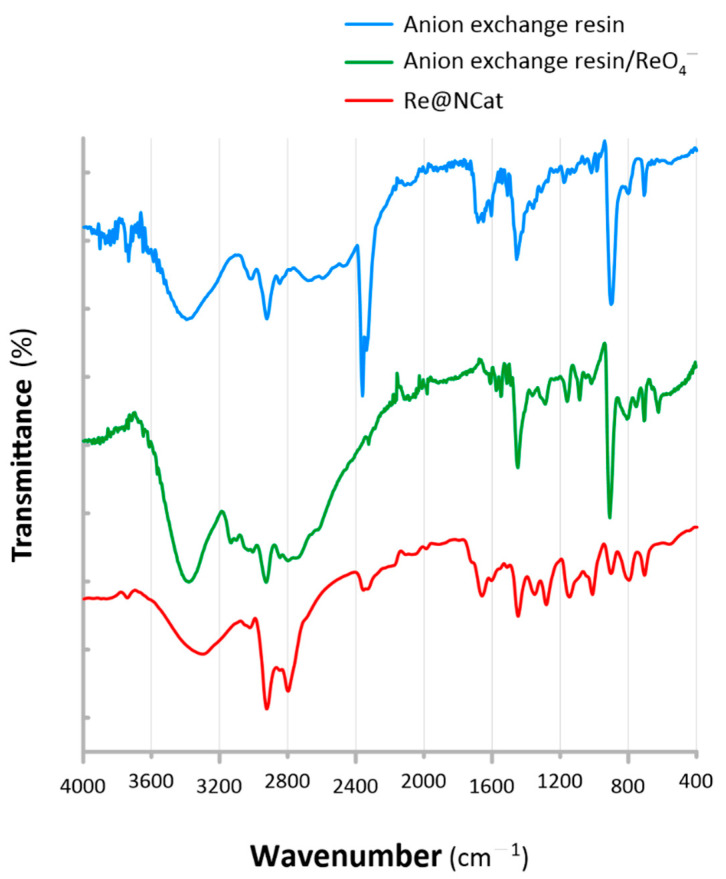
ATR FT-IR spectra recorded for the anion exchange resin (CIM-functionalized VBC-co-DVB copolymer), the ReO_4_^−^ loaded anion exchange resin, and the resultant Re@NCat.

According to the route displayed in Figure 1, synthesis of the Re@NCat involved at first the production of anion exchange resins containing functionalities derived from CIM. Afterwards, the relevant polymer was employed to adsorb ReO_4_^−^ ions, and it was further reduced on amino ligands by NaBH_4_. To verify this protocol, each stage of the above-mentioned synthesis was monitored by recording ATR FT-IR spectra. The collected data are displayed in Figure 2.

The polymeric base used for synthesis of the Re-based nanocomposite (CIM-functionalized copolymer) revealed the presence of a series of bands attributed to the amino ligands derived from CIM. This included vibration bands at 3394 cm^−1^, 1540 cm^−1^, and 1357 cm^−1^ attributed, respectively, to the N–H stretching, the C=N aromatic ring deformation, and the N–C–N stretching (Figure 2, Table 1) [28]. All these identified moieties were consistent with the structure of CIM (see Figure 1) and pointed out that the relevant anion exchange resin was successfully synthesized. The ATR FT-IR spectrum of the CIM-functionalized VBC-co-DVB copolymer also indicated that the CIM functionality was attached to the polymeric matrix. Accordingly, C=O stretching vibrations at 1681 cm^−1^, as well as at 1648 cm^−1^ for R–N–CO–N–R bonds were identified as well (see Figure 2). Simultaneously, the presence of C–O–C stretching vibrations at 1143, 1014, and 906 cm^−1^ and N^+^–H aromatic stretching vibration at 2472 cm^−1^ [28] were noted. This indicated that the CIM functionality was likely attached to the polymeric matrix, by either the –N^+^– moiety in the aromatic ring as well as through the C=O group. The latter one was selected to be displayed in Figure 1 as a simplified representation.

Then, FT-IR analysis was repeated for the CIM-functionalized resin loaded with ReO_4_^–^ ions, and again when oxyanions were reduced, leading to synthesis of the Re@NCat. The data indicating changes in the resin’s structure are summarized in Table 1. After adsorption of ReO_4_^–^ ions on the CIM functionality (see Section 2.2 for details), the above-discussed bands (attributed to the amino group) gradually shifted (see Table 1). After reduction of oxyanions, these shifts further increased their magnitude to achieve the effect in which the following transitions were observed: from 3394 to 3297 cm^−1^ (a 97 cm^−1^ shift), from 1540 to 1546 cm^−1^ (a 6 cm^−1^ shift), from 1648 to 1658 cm^−1^ (a 10 cm^−1^ shift), and from 1357 to 1349 cm^−1^ (8 cm^−1^ shift). This suggested a primary role of the CIM functionality in the capping and stabilization of the resultant ReNSs [29,30]. It was also noticed that the vibration band at 2472 cm^−1^, attributed to the N^+^–H aromatic stretching, faded, and a new one at 2796 cm^−1^, attributed to the H–bridge in the NH chelating group, appeared. This suggested the chelation of ReO_4_^–^ ions on CIM through both amino and carbonyl functionalities [31,32].

These results proved that the synthesis of the polymeric base was successful. In addition, they proved that ReNSs were effectively formed and stabilized with the aid of the CIM functionality.

### 3.2. Loading the Anion Exchange Resin with the ReNSs

After completing the anion exchange resin followed by the reduction of ReO_4_^−^ ions, the anion exchange resin changed its color from yellow to black. This, parallel with shifts of vibration bands associated with amino functionalities (see the previous section), suggested the stabilization of ReNSs in the polymeric matrix. Simultaneously, the concentration of Re in the resultant Re@NCat was 10.0 ± 0.5 mg g^−1^, i.e., ≈1% (*w*/*w*) per catalyst mass (based on the ICP-OES analysis of this material).

**Figure 3:**
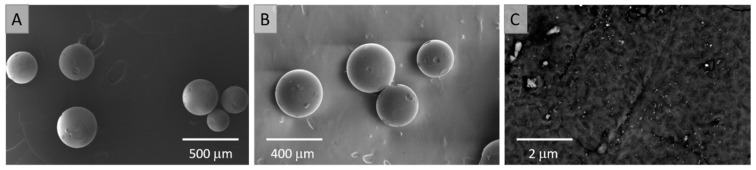
SEM photomicrographs of the Re@NCat sample. (**A**) The CIM-functionalized anion exchange resin, (**B**) the Re@NCat, (**C**) the surface morphology of the Re@NCat.

The morphology of the resultant Re@NCat was firstly investigated using the SEM imaging. The acquired photomicrographs are displayed in Figure 3.

As can be seen in Figure 3A,B, synthesis of the VBC-co-DVB suspension copolymer was successful. The obtained resin is characterized by the spherical shape morphology, with the size ranging from 0.2 to 0.4 mm. Since the VBC-co-DVB copolymer was obtained in the presence of toluene (a good solvent for monomers), the synthesized resin was characterized by the expanded gel structure. This was reflected by the apparent smooth surface of the grains (Figure 3A,B) that after magnification (Figure 3C) revealed a complex nature attributed to the swelling of polymeric chains in toluene [33,34]. Simultaneously, Figure 3C displays the photomicrograph captured using the back scattered electrons (BSE) technique that revealed a clear phase contrast between the polymeric matrix (dark area) and ReNSs (bright spots). This indicated that ReNSs were indeed incorporated into the structure of the anion exchange resin. In parallel, adsorption-driven synthesis provided a proper dispersion of ReNSs. However, due to the apparent size of ReNSs, the SEM analysis was not sufficient to investigate them. Therefore, to evaluate the morphology of the resultant ReNSs, the Re@NCat sample was cut into ultra-thin slices and was subjected to the TEM analysis.

**Figure 4:**
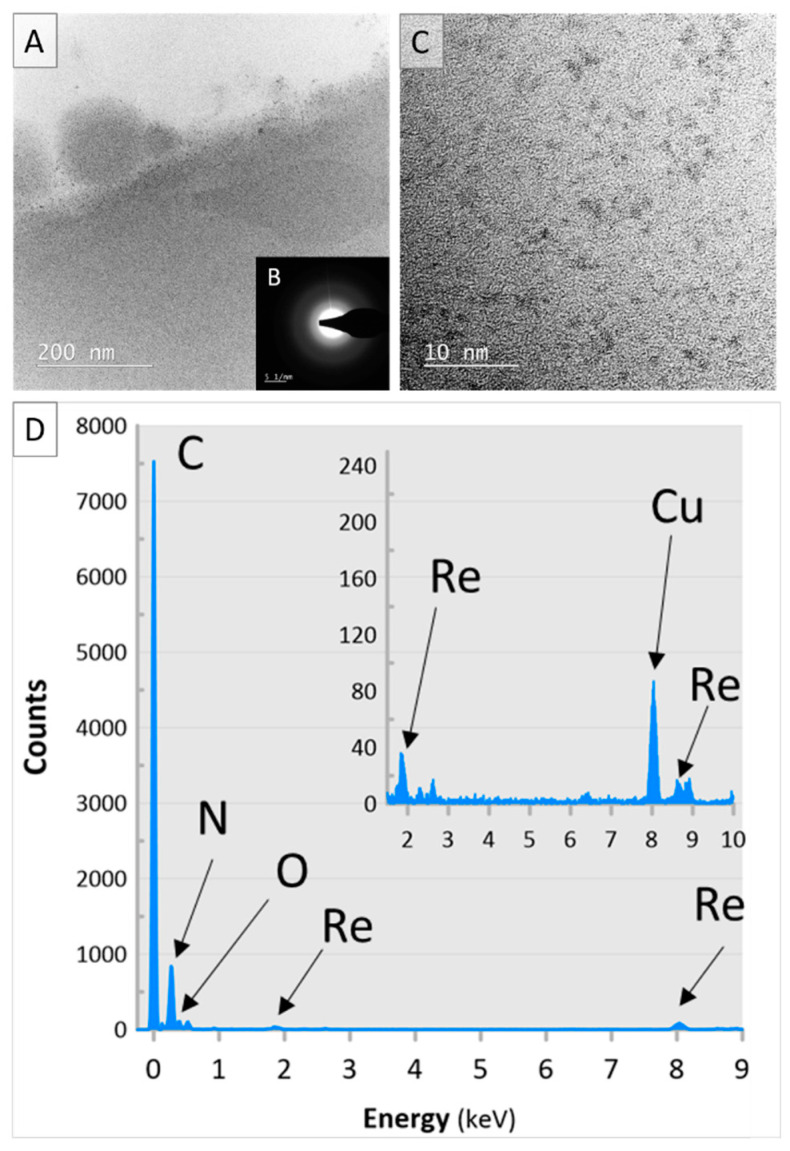
(**A**,**C**) TEM photomicrographs supplied with (**B**) SAED and (**D**) EDX spectra of the Re@NCat sample. The inset in panel D is a magnification of the EDX spectrum recorded in the range of 2–10 keV.

In reference to TEM photomicrographs (Figure 4A,C), it appeared that the applied synthetic route resulted in a successful fabrication of spherical in shape ReNSs and their loading in the polymeric matrix. Generally, diameters of synthesized ReNSs spanned the range of 1–15 nm. However, larger structures (10–15 nm in size) were also noted near the surface of the Re@NCat (Figure 4A), while the inner part of the polymer’s grain contained much smaller particles (1–3 nm) or their agglomerates (≈5 nm) (Figure 4C). This effect was predicted as all previous observations proved that the average size of NSs obtained using this method strongly depends on the concentration gradient at the solid–liquid interface [15,29]. Nevertheless, the average size of ReNSs found inside the polymer’s grain was 3.45 ± 1.85 nm.

The EDX spectrum (see Figure 4D) clearly showed that investigated NPs were based on Re. However, the applied synthetic route involved a reduction of ReO_4_^−^ ions with Re at the +7 oxidation state. Therefore, the formation of NPs of Re at various oxidation states was possible. In the presence of atmospheric O_2_, it could include mainly oxides, such as ReO_2_ and ReO_3_ with Re^4+^ and Re^6+^ [26,35,36]. To limit this possibility, the in situ production procedure of ReNSs (see Section 3.1 for details) was carried out using the reaction media purged with N_2_. This approach resulted in the fabrication of ReNSs, in which the SAED pattern (Figure 4B) revealed their amorphous structure, which was likely due to the appearance of Re–Re bonds [37,38,39]. Based on these observations, it was concluded that the produced ReNSs were indeed ReNPs with the Re^0^ form.

### 3.3. Catalytic Hydrogenation of Nitroaromatics

**Figure 5:**
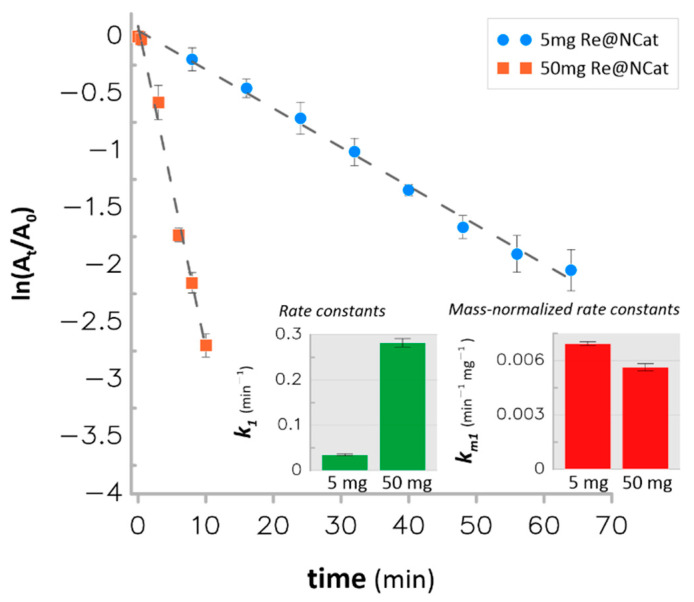
First-order kinetics plots for the catalytic hydrogenation of 4-NP using 5 and 50 mg of the Re@NCat.

The synthesized Re@NCat material was used as a new heterogeneous catalyst for the hydrogenation of 4-NP and 4-NA. First, the catalyst mass taken for these tests was optimized. To achieve thereof, 5 and 50 mg of the Re@NCat were used for 4-NP reduction. In parallel, the same masses of the anion exchange resin (without ReO_4_^−^ ions or ReNSs) were tested for the same catalytic reactions as appropriate controls. Figure 5 displays pseudo-first-order kinetics plots for this process.

Based on collected UV-Vis spectra (results not shown here), the anion exchange resin itself was established to be unable to catalyze both studied reactions because no effect at all, even at prolonged contact times with 4-NP and 4-NA, was observed. Then, it was established that the Re@NCat material led to 87–94% hydrogenation of 4-NP within 64 min when its 5 mg was applied and within 10 min when its 50 mg was used. Simultaneously, pseudo-first-order rate constants (*k*_1_) assessed in these conditions were 0.035 and 0.282 min^−1^ for 5 and 50 mg of the Re@NCat, respectively (Figure 5). The greater mass of the Re@NCat consequently led to the greater *k*_1_ value; however, the actual catalyst efficiency was confirmed by the value of the mass-normalized rate constant (*k_m_*_1_) that represents the catalyst activity with respect to its mass applied within the process. In this context, while 50 mg of the Re@NCat had the *k_m_*_1_ *=* 5.63 × 10^−3^ min^−1^ mg^−1^, its 5 mg portion revealed the outstanding *k_m_*_1_ *=* 6.94 × 10^−3^ min^−1^ mg^−1^.

At the moment, there is no other example of the Re-based heterogeneous NCat that was applied for the hydrogenation of NARs. However, to the best of our knowledge, there is one report focusing on homogenous catalysts based on ReNPs that were used for the catalytic hydrogenation of 4-NP [26]. In this particular case, ReNPs were loaded into highly porous carbon nanostructures and revealed a greater *k*_1_ value for the catalytic reduction of 4-NP, i.e., 8.99 min^−1^, vs. 0.282 min^−1^ obtained in the present work. However, it must be noticed that in the latter case, the concentration of Re in the synthesized heterogeneous Re@NCat was at a trace level (≈1% (*w*/*w*). In this context, it can be recognized as a more economically viable material along with its convenience arising from heterogeneous catalysis, i.e., its ease of operation and stability, which will be highlighted further in the manuscript.

Comparing the Re@NCat with other recently reported heterogeneous NCats with AuNPs, the obtained *k_m_*_1_ value of 4-NP hydrogenation was higher as compared to those assessed for AuNPs loaded on a mung bean starch (*k_m_*_1_ = 0.4 × 10^−3^ min^−1^ mg^−1^) [40] or an amino-functionalized olive biomass (*k_m_*_1_ = 5.7 × 10^−3^ min^−1^ mg^−1^). Although Re does not formally belong to PGMs, its ionic properties (ability to form stable oxoanions) allow grouping it with these metals. As such, the obtained *k*_1_ values are much greater than those assessed for a PdNPs homogenous NCat (*k*_1_ = 0.0125 min^−1^) [41], or NCats being Pt–Au and Pt dendrimers immobilized on graphene oxide (*k*_1_ = 0.041 min^−1^ and 0.057 min^−1^, respectively) [42].

**Figure 6:**
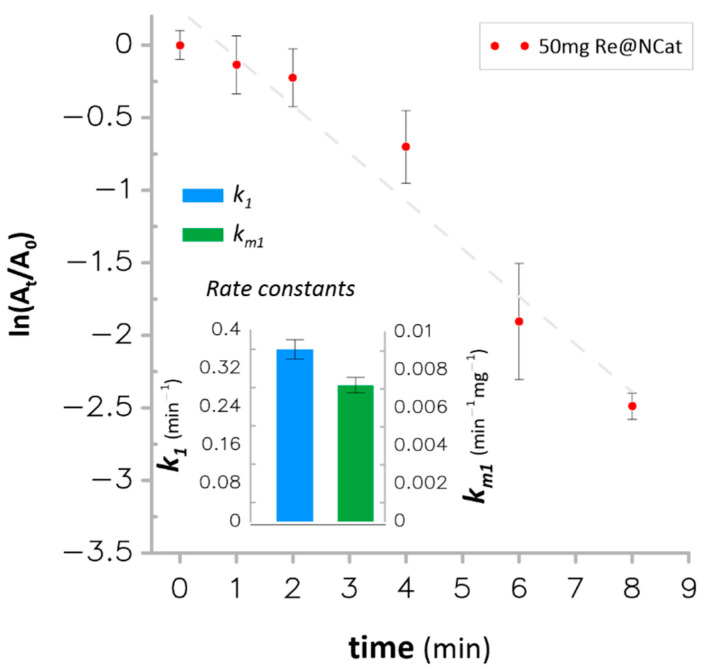
The first-order kinetics plot for catalytic hydrogenation of 4-NA using 50 mg of the Re@NCat.

The catalytic reduction of 4-NP was considered as a model reaction, which is convenient for assessing the catalyst activity of the new Re@NCat. Another reaction that was selected, although much less popular, was the catalytic reduction of 4-NA to 1,4-phenylenediamine [43,44]. Therefore, the Re@NCat was also applied for the catalytic hydrogenation of 4-NA. Since 50 mg of the catalyst was responsible for the fastest reduction of 4-NP, it was also applied in the present process. The obtained pseudo-first order plot is displayed in Figure 6.

The Re@NCat led to a >90% reduction of 4-NA within 8 min with the apparent rate constant *k*_1_ = 0.360 min^−1^ and the mass normalized rate constant *k_m_*_1_ = 7.2 × 10^−3^ min^−1^ mg^−1^. These values were approximately 20% greater as compared to the corresponding rate constants obtained for the catalytic hydrogenation of 4-NP. However, they were smaller than those obtained for the homogenous catalyst containing ReNPs loaded onto carbon nanostructures (*k*_1_ = 8.72 min^−1^) [26]. However, the heterogeneous Re@NCat material offered more facilitated 4-NA hydrogenation as compared to polymer-encapsulated ReNPs used as the homogenous catalyst for this process [25]. In this case, although it was expected that the heterogeneous Re@NCat should have smaller *k*_1_ values, it outperformed the large size (≈1.7 nm) and small size (≈0.7 nm) ReNPs that led to reduction of 4-NA with rate constants *k*_1_ = 0.152 and 0.0758 min^−1^, respectively [25]. When considering PGMs NPs, the Re@NCat had also the greater catalytic activity compared to that found for the trimetallic FeAgPt homogenous catalyst, i.e., the rate constant *k*_1_ = 0.1 min^−1^ for 4-NA reduction [45]. The Re@NCat revealed the greater catalytic activity in the case of 4-NA reduction when also compared to other metallic NPs, which were recently reported as heterogeneous NCats. This includes AgNPs loaded into crosslinked polymeric hydrogels (*k*_1_ = 0.0115–0.4692 min^−1^) [46] and AgNPs immobilized onto PU/keratin nanofibrous mats (*k*_1_ = 0.072–0.114 min^−1^) [47].

**Figure 7:**
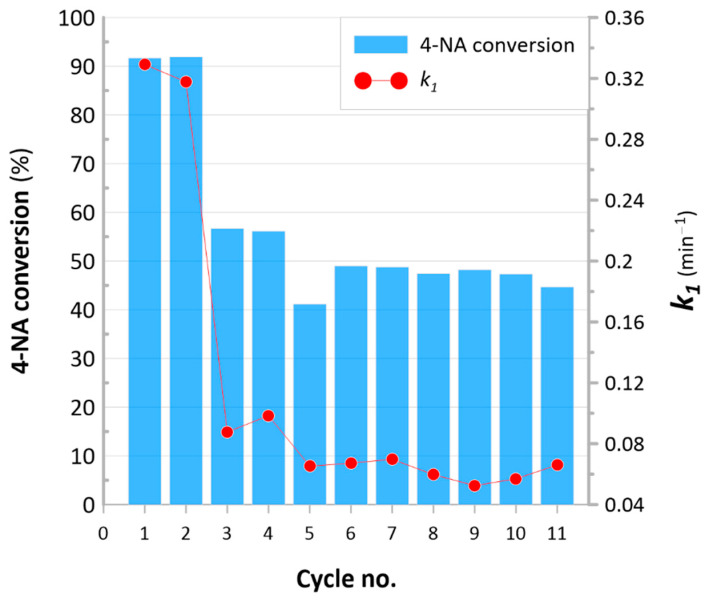
First-order rate constants (*k*_1_) and maximum conversion values of 4-NA to 1,4- phenylenediamine within 8 min and using 50 mg of the Re@NCat.

Based on these results and the literature review, it was established that the Re-based heterogeneous Re@NCat revealed the superior catalytic activity in the hydrogenation of 4-NP and 4-NA, even though the concentration of active sites (Re) in the polymer was ≈1%. This suggests a prospective use of nanomaterials containing ReNPs in different catalytic systems. Despite the price of Re, its catalytic activity linked with its trace amounts required to achieve the desired effect (efficient reduction of NARs), it appears that the Re@NCat could be a real alternative toward already reported solutions. The synthesized NCat showed yet another significant advantage. Its morphology, arising from the polymeric matrix (the anion exchange resin based on the suspension copolymer), enabled easily separating and re-using the catalytic material [15]. Hence, the Re@NCat was used in the catalytic reduction of 4-NA over 11 subsequent catalytic cycles, within which the maximum conversion in time defined as 8 min and the *k*_1_ value were estimated (see Figure 7).

Based on these results, it could be stated that the initial catalytic activity (>90% of 4-NA conversion, *k*_1_ ≅ 0.32 min^−1^) was retained over two initial catalytic cycles. After this, the reduction yield (%) dropped and remained in the range of 44–56%. Simultaneously, values of the first-order rate constant (*k*_1_) decreased. After the initial period (two cycles), *k*_1_ values were in the range of 0.57–0.88 min^−1^ (Figure 7). Although efficiency of the Re@NCat decreased over the cycle number, it must be remembered that to re-use it, it had to be just simply filtrated and directly re-introduced into a next run of the tested catalytic reaction. This makes the proposed NCat unique among others that often require (when it is possible) supplying intensive methods for their separation [48]. Despite this, minimal *k*_1_ values obtained for an 8-min reduction of 4-NA (Figure 7) are still better than those achieved for the other NCats discussed and compared above.

However, it is interesting that similar NCats based on anion exchange resins but containing AuNPs retained their catalytic activity over 11 catalytic runs of the studied catalytic reduction of 4-NP [15]. To explain this phenomenon, our attention was initially drawn to differences in the structure of 4-NA and 4-NP. The working hypothesis was that the drop after the second catalytic cycle could be due to a hindered release of resultant 1,4-phenyldiamine from the polymer’s structure. In this scenario, it could be concluded that the decreased adsorption capacity of the polymeric matrix could effectively decrease the catalytic activity of the Re@NCat in the following cycles. However, after examining collected UV-Vis spectra (results not shown here), it was found that catalytic reaction resulted in a gradual decrease in the absorption band at ≈300 nm attributed to 4-NA reduction. This phenomenon disproved the above-stated hypothesis. The other reason for decreasing the catalytic activity of the Re@NCat could be a possible leaching of Re from the polymeric matrix. To verify this possibility, solutions remaining after catalytic reactions were tested toward their ability to scatter the light, and on the presence of Re, which was verified using ICP-OES. There was no scattering of the light by these solutions and they contained no Re, which excluded the possibility of the ReNPs leaching.

**Figure 8:**
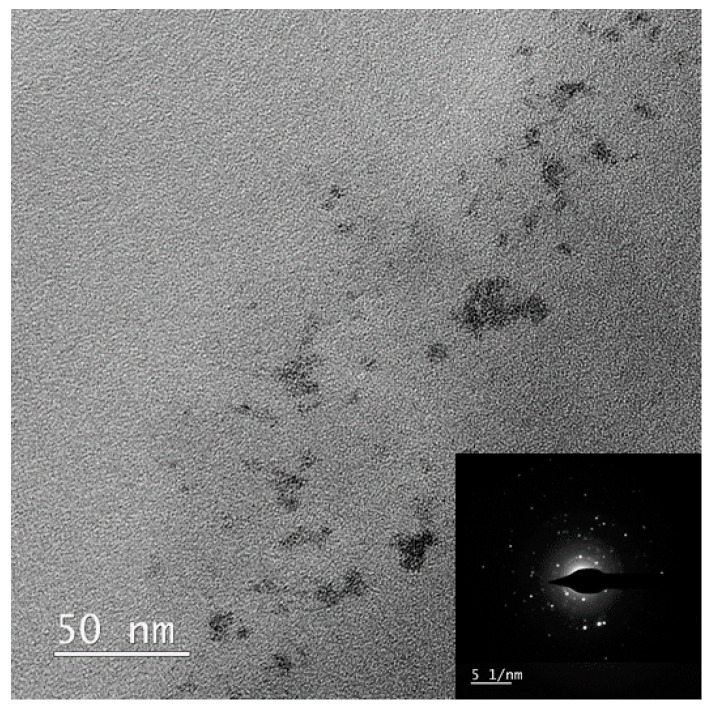
The TEM photomicrograph of the Re@NCat after the 11th catalytic run.

The actual reason for the decreased catalytic activity of the Re@NSs could be concluded based on mutual relations between yields of 4-NA conversion (%) and *k*_1_ values (Figure 7). After the second catalytic reaction, both of these values dropped and remained relatively constant over further subsequent reactions (up to the 11th cycle). This suggests that ReNPs present in the Re@NCat could be transformed. It was reported before [26] that ReNPs are susceptible to interact with O_2_ (both atmospheric and dissolved in H_2_O), forming oxygen-doped ReNSs. If so, the prolonged exposure of the Re@NCat to the H_2_O-based, open-to-atmosphere air environment could be the reason for a change in its catalytic activity. To verify this conclusion, the Re@NCat collected at the end of the experiment (after the 11th run of the catalytic reaction) was subjected to the TEM analysis. The corresponding photomicrograph with the SAED pattern is displayed in Figure 8.

As can be seen, there was indeed a difference between the Re@NCat before use (Figure 4) and the used one (Figure 8). In the latter case, more larger structures (>10 nm) were observed (Figure 8), which suggested a partial agglomeration of ReNPs. However, more importantly, the displayed SAED pattern (Figure 8) was significantly different as compared to the one acquired for the virgin Re@NCat (Figure 4B). As stated above, the lack of reflections visible in Figure 4B suggested the presence of ReNSs with Re^0^ [37,38,39]. On the contrary, the pattern observed in Figure 8 contained a series of reflections. This major difference suggests the presence of oxide-doped ReNSs [37,38,39] in the Re@NCat after running all subsequent catalytic reactions. This may also point out that ReNSs in the Re@NCat were indeed oxidized during its use, explaining why its initial catalytic activity dropped and remained constant until the end of the experiment.

## 4. Conclusions

In this work, we propose a new NCat based on the polymeric nanocomposite consisting of the anion exchange resin and ReNPs. Its synthesis involved the reduction-coupled adsorption process of ReO_4_^−^ ions and resulted in fabricating spherical ReNPs, having the average size of 3.45 ± 1.85 nm and being further stabilized by the amino functionality derived from 1,1′-carbonyldimidazole in the polymeric matrix.

Then, the so-obtained Re@NCat was used in the catalytic reduction of NARs, i.e., 4-NP and 4-NA. It outperformed other heterogeneous and some homogenous NCats containing AuNPs as well as NPs of PGMs. This indicated the primary role of unique and rare ReNPs in the facilitation of catalytic hydrogenation of NARs. The Re@NCat was also found to be suitable for the re-use in reduction of 4-NA. The catalytic activity thereof was retained over two subsequent catalytic cycles, after which an approximately two-fold decrease was noted. After this, the catalytic activity remained constant up to the 11th catalytic cycle. The observed initial decrease in the catalytic activity could be attributed to the susceptibility of the resultant ReNPs to oxidation-driven passivation, which was likely caused by O_2_ present in the open-to-air atmosphere and/or in H_2_O-based environment.

Based on this, the heterogeneous polymeric NCat containing ReNPs can be considered as a prospective and very tempting alternative to solve the reported catalytic problems in the hydrogenation of NARs. However, due to its unique character and rare occurrence in the literature, Re-based NCats still need more studies to confirm their unique and phenomenal catalytic properties and take the full advantage of them. For example, it should be answered whether is it possible to push the catalytic activity of these materials further by adjusting their granulometric properties. Such future research should also answer the question to what extent polymer-stabilized ReNPs should be isolated from the influence of the surrounding atmosphere.

## Figures and Tables

**Table 1:** The location of FT-IR bands found in the anion exchange resin (polymeric base) before and after sorption of ReO_4_^-^ ions, as well as in the resulting Re@NCat.

Interactions	Characteristic Band Location/Shift (cm^−1^)				
	A-X Resin		A-X Resin + ReO_4_^−^		Re@NCat
N–H stretching	3394		3369	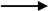	3297
C=N aromatic deformation	1540	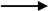	1544	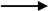	1546
R–N–CO–N–R stretching	1648	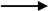	1649	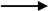	1658
N–C–N stretching	1357	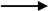	1359	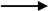	1349
N^+^–H aromatic stretching	2472	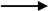	2454	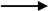	-
H–bridge	-	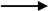	2794	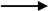	2796

A-X: anion exchange.

## Data Availability

Data is contained within the article.
